# Adult-Onset Still's Disease and Pulmonary Embolism: A Case Report

**DOI:** 10.7759/cureus.69245

**Published:** 2024-09-12

**Authors:** May A Alotaibie, Mohammed Alqahtani, Siraj Rajendram

**Affiliations:** 1 Neurology, National Guard Health Affairs, Riyadh, SAU; 2 Internal Medicine, National Guard Health Affairs, Riyadh, SAU

**Keywords:** adult onset still disease, anticoagulation, apixaban, hypercoagulability, pulmonary embolism, venous thromboembolism

## Abstract

Adult-onset Still's disease (AOSD) is a rare systemic inflammatory disorder characterized by fever, rash, arthralgia, and systemic inflammation. Pulmonary embolism (PE), a potentially life-threatening complication, is infrequently associated with AOSD. This report presents a unique case that highlights the importance of considering atypical presentations of PE in this patient population despite the absence of classical risk factors. An 84-year-old male with hypertension, benign prostatic hypertrophy, and AOSD diagnosed six years prior, presented with confusion, fever, and malaise for two days. He denied any recent travel or immobilization. Examination revealed tachycardia and reduced oxygen saturation on room air. D-dimer was elevated, and CT chest angiography (CTCA) confirmed left upper and lower segmental PE without cardiac strain. Investigations for infection were negative. Initial treatment with intravenous heparin was switched to apixaban. The patient was eventually discharged home with stable vital signs.

The inflammatory state in AOSD might contribute to hypercoagulability, increasing the risk of PE. This case emphasizes the importance of considering PE in AOSD patients, even in the absence of classical risk factors, to prevent potentially fatal complications. A literature review revealed few cases of AOSD presenting with PE, highlighting the atypical presentation and need for increased awareness. This case underscores the rare, but potentially serious, association between AOSD and unprovoked PE. Clinicians managing AOSD should maintain a high index of suspicion for PE, particularly in patients presenting with unexplained respiratory symptoms.

## Introduction

Adult-onset Still’s disease (AOSD) is a rare systemic inflammatory disorder characterized by a range of clinical features [1]. While its etiology remains elusive, it probably involves a complex interplay of genetic and environmental factors [2]. Yamaguchi’s criteria remain the cornerstone in diagnosing AOSD. These criteria include a combination of clinical and laboratory findings [3,4]. Pulmonary embolism (PE), a potentially life-threatening complication characterized by arterial occlusion, is a leading cause of morbidity and mortality [5,6]. This case report describes the presentation of an unprovoked PE in a patient with established AOSD, highlighting the critical need to consider PE in this population despite the absence of conventional risk factors.

## Case presentation

An 84-year-old male with a medical history of hypertension, benign prostatic hypertrophy, and chronic AOSD diagnosed in 2017 (treated with methotrexate 10 mg weekly, folic acid six days a week, and prednisolone 5 mg daily), presented with a two-day history of fever, malaise, and confusion. He reported contact with a sick individual. The initial vital signs revealed tachycardia (110/minute) and reduced oxygen saturations (88% on room air), necessitating the administration of supplemental oxygen (2 liters/minute via nasal cannula). Physical examination was unremarkable but laboratory blood tests revealed a white blood cell (WBC) count of 12.5 x10^9^/L, D-dimer of 23.6 mg/L, and troponin I of 72 ng/dl (Table 1).

**Table 1:** Lab investigations

Variable	Patient value	Normal range
White blood cells	12.5	4-11 /L
D-dimer	23.67	0-0.5 mg/L
Troponin I	72	0-23 pg/ml

**Figure 1:**
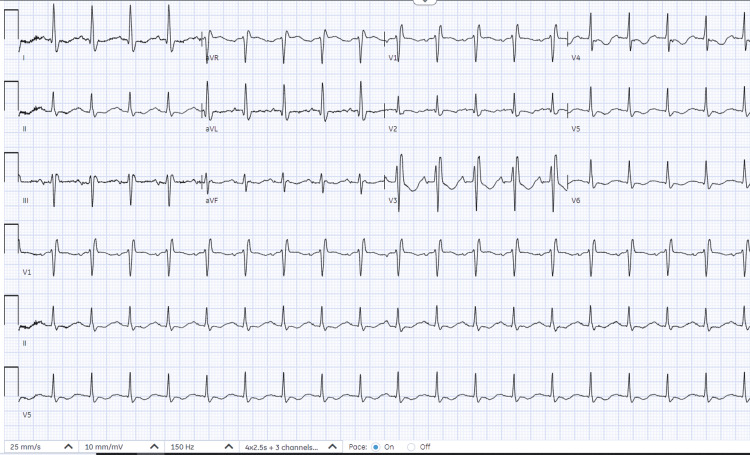
Electrocardiography Electrocardiography showed sinus rhythm with a heart rate of 120 beats per minute

**Figure 2:**
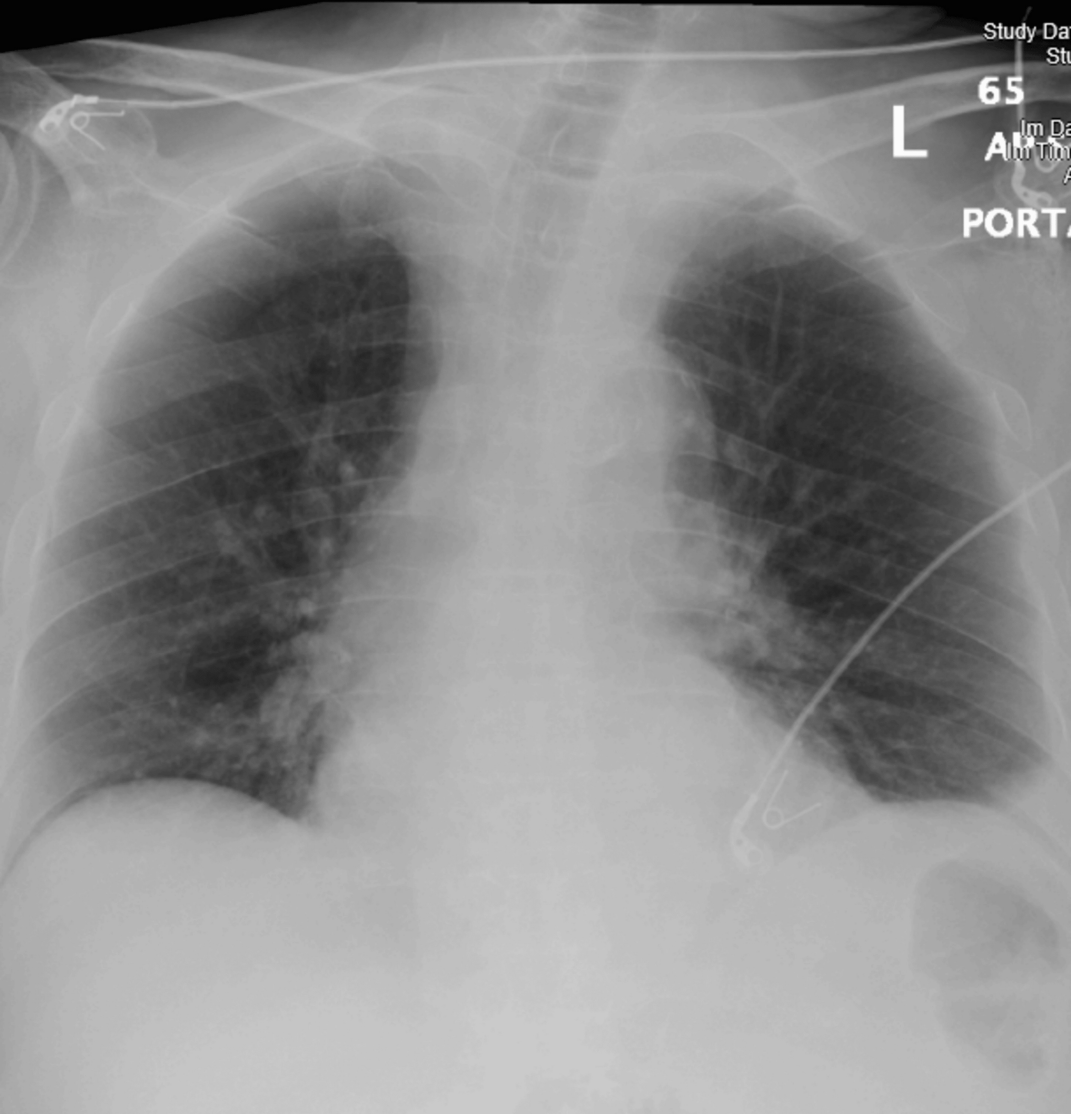
Chest X-ray Chest X-ray revealed non-significant changes, no clear opacity, and no regional oligemia

**Figure 3:**
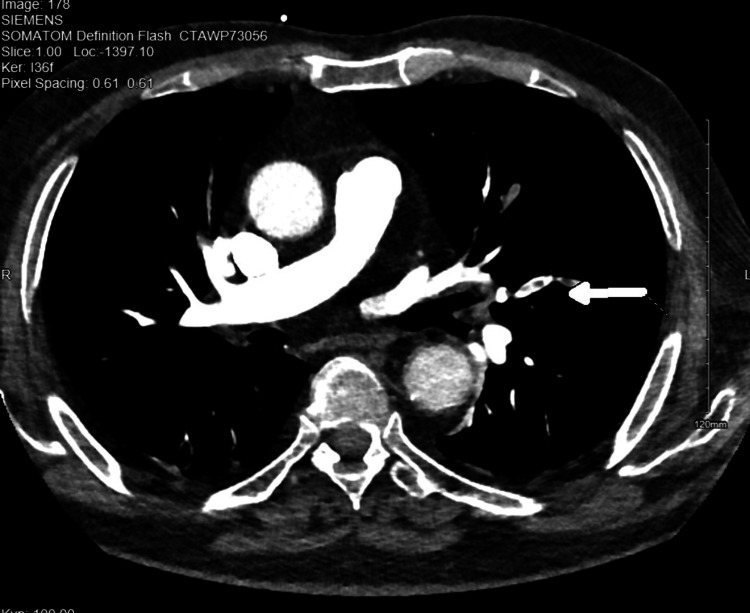
CTCA CTCA revealed upper and lower segmental pulmonary embolism without evidence of cardiac strain CTCA: computed tomography chest angiography

Electrocardiography demonstrated sinus tachycardia (Figure 1). Although chest X-ray was unremarkable (Figure 2), CT chest angiography (CTCA; Figure 3) revealed left upper and lower segmental PE without evidence of cardiac strain. All infectious workups were negative. Treatment commenced with intravenous heparin, and the patient’s clinical condition and vital signs improved. The patient was subsequently discharged on apixaban (10 mg twice daily for seven days, followed by 2.5 mg twice daily).

## Discussion

The diagnosis of AOSD, characterized by multi-organ involvement, necessitates excluding infectious, malignant, and other autoimmune etiologies [1]. Yamaguchi's criteria, boasting a sensitivity of 93.9%, guide the diagnosis of AOSD [4]. These criteria require at least five points, including two major criteria (fever exceeding 39 °C for one week, arthralgia lasting at least two weeks, characteristic rash, and elevated WBC count with predominant granulocytes). Minor criteria include liver dysfunction, sore throat, lymphadenopathy/splenomegaly, and negative rheumatoid factor and antinuclear antibodies [7]. Our patient fulfilled three major and two minor criteria, leading to the diagnosis of AOSD after the exclusion of alternative etiologies. The clinical course of AOSD follows one of three typical patterns: monophasic (self-limiting), intermittent (characterized by flares and remissions), and chronic (predominantly with variable joint involvement) [7].

End-organ damage associated with AOSD includes cardiovascular complications, amyloidosis, renal impairment, fulminant hepatitis, and hematologic complications [e.g., microangiopathic hemolytic anemia, thrombotic thrombocytopenic purpura, disseminated intravascular coagulation, and, as in the present case, unprovoked venous thromboembolism (VTE)] [8,9]. PE can manifest with dyspnea, chest pain, syncope, and less frequently, fever, hemoptysis, cyanosis, or shock [10]. Evaluation for PE relies on risk stratification. D-dimer testing holds high sensitivity for excluding PE in low-risk patients [10].

CTCA, which is considered the most accurate non-invasive diagnostic modality for PE, is typically requested for high-risk patients and those with positive D-dimer results [10]. Our patient's presentation with fever and dyspnea, coupled with elevated D-dimer, prompted a CTCA that confirmed PE. Of note, this case lacked traditional PE risk factors besides AOSD. The association between AOSD and PE is uncommon. However, a cohort study in Oman has reported a concerning prevalence of fatal PE (16.7%) in their AOSD population, suggesting that VTE may not be as rare as previously thought [11]. Published cases have highlighted instances of unprovoked PE in AOSD patients with elevated ferritin levels, potentially reflecting the underlying inflammatory process characteristic of AOSD [9].

Veronica et al. have described a 56-year-old Caucasian patient with AOSD who presented with bilateral lower limb edema and dyspnea. The D-dimer was elevated, lower limbs ultrasound showed extensive deep vein thrombosis (DVT), and CTCA revealed bilateral pulmonary embolism [12]. Another case reported in 2021 involved a middle-aged man with AOSD who initially presented with limb swelling. Ultrasound confirmed extensive DVT and, despite anticoagulation with apixaban, the patient developed recurrent DVT and non-occlusive PE four weeks later [13]. Moreover, AOSD may present with PE. For example, Bhamra et al. have reported a case of a 66-year-old woman initially presenting with respiratory symptoms due to segmental PE without provoking factors, followed by the development of a rash, arthralgia, and myalgia ultimately leading to the diagnosis of AOSD [14].

These cases emphasize the potential for AOSD to predispose patients to VTE, and underscore the importance of considering AOSD as a potential risk factor for VTE. Hence, a high index of suspicion for VTE must be maintained when encountering patients with AOSD, even in the absence of conventional risk factors. Early diagnosis and intervention are paramount to prevent potentially life-threatening complications in this patient population.

## Conclusions

This report explored a unique case of unprovoked PE in an elderly patient with established AOSD. It underscores the importance of considering PE as a potential complication in AOSD patients, even in the absence of traditional risk factors. Clinicians managing AOSD patients should maintain a heightened awareness for PE, particularly in those presenting with unexplained respiratory symptoms or other suggestive clinical features. Further research is warranted to elucidate the underlying mechanisms linking AOSD to VTE and optimize risk stratification strategies for this patient population.
